# Effects of Mechanical Ball Milling Time on the Microstructure and Mechanical Properties of Mo_2_NiB_2_-Ni Cermets

**DOI:** 10.3390/ma12121926

**Published:** 2019-06-14

**Authors:** Lei Zhang, Zhifu Huang, Yangzhen Liu, Yupeng Shen, Kemin Li, Zhen Cao, Zijun Ren, Yongxin Jian

**Affiliations:** 1 State Key Laboratory for Mechanical Behavior of Materials, Xi’an Jiaotong University, Xi’an 710049, China; xjtulei@outlook.com (L.Z.); ilovehit3@126.com (Y.S.); kmlixjtu@outlook.com (K.L.); zcao_xjtu@163.com (Z.C.); 2 Institute of Advance Wear & Corrosion Resistant and Functional Materials, Jinan University, Jinan 510632, China; liuyangzhen626@163.com; 3 Instrumental Analysis Center of Xi’an Jiaotong University, Xi’an 710049, China; renzijun@xjtu.edu.cn

**Keywords:** powder metallurgy, ball milling, Mo_2_NiB_2_-Ni cermets, microstructure, mechanical properties

## Abstract

Mo_2_NiB_2_-Ni cermets have been extensively investigated due to their outstanding properties. However, studies have not systematically examined the effect of the powder milling process on the cermets. In this study, Mo, Ni, and B raw powders were subjected to mechanical ball milling from 1 h to 15 h. XRD patterns of the milled powders confirmed that a new phase was not observed at milling times of 1 h to 15 h. With the increase in the mechanical ball milling time from 1 h to 11 h, raw powders were crushed to small fragments, in addition to a more uniform distribution, and with the increase in the mechanical ball milling time to greater than 11 h, milled powders changed slightly. Mo_2_NiB_2_-Ni cermets were fabricated by reaction boronizing sintering using the milled powders at different ball milling times. The milling time significantly affected the microstructure and mechanical properties of Mo_2_NiB_2_-Ni cermets. Moreover, the Mo_2_NiB_2_ cermet powder subjected to a milling time of 11 h exhibited the finest crystal size and the maximum volume fraction of the Mo_2_NiB_2_ hard phase. Furthermore, the cermets with a milling time of 11 h exhibited a maximum hardness and bending strength of 87.6 HRA and 1367.3 MPa, respectively.

## 1. Introduction

Mo_2_FeB_2_-, Mo_2_NiB_2_-, and WCoB-based cermets have been successfully fabricated by reaction boronizing sintering [1,2,3,4], generating a ternary boride hard phase by liquid-phase sintering. Boride cermets can be prepared by using a metal binder phase. These cermets exhibit outstanding mechanical properties, including high hardness, good bending strength, and excellent fracture toughness. In particular, Mo_2_NiB_2_-Ni cermets [5,6], comprising the Mo_2_NiB_2_ hard phase that contributes to the hardness and the Ni binder phase that contributes to the toughness. These cermets have attracted considerable attention due to their excellent abrasion resistance, anti-oxidation properties, and superior corrosion resistance [7,8]. With those unique properties, Mo_2_NiB_2_-Ni cermets exhibit good prospects for engineering materials in wear- and erosion-resistant applications, and cermets are expected to replace conventional materials, including nickel-based alloys in injection molding machine parts and copper alloys in pumps used in oil extraction and transport.

Previous studies of Mo_2_NiB_2_-Ni cermets have focused on the improvement of mechanical properties. A series work of Takagi et al. [9] has reported the effect of element doping on the microstructure and mechanical properties of Mo_2_NiB_2_-Ni cermets. Their study revealed that the addition of Cr and V elements can render remarkable strength to the solid solution by the dissolution of the Mo_2_NiB_2_ hard phase. Hence, the mechanical properties of Mo_2_NiB_2_-Ni cermets, such as hardness and fracture toughness, can be significantly improved by the addition of Cr and V elements, which refines the grains of Mo_2_NiB_2_. However, the addition of other elements, e.g., Fe, Co, Ti, and Mn, can reduce the performance of the cermets [10,11], mainly related to the contamination of grain boundaries. Furthermore, Takagi et al. [12] have revealed that the properties of bulk Mo_2_NiB_2_ can be improved by the adjustment of Mo/B due to the change in the Mo_2_NiB_2_ cell lattice. However, marginal attention has been focused on the fabrication of Mo_2_NiB_2_-Ni cermets. In particular, a systematic investigation focusing on the powder milling process has not been reported.

For powder metallurgy, planetary ball milling is typically employed for preparing milled powders due to its convenience and cost-effectiveness. During milling, to achieve the optimization of milled powders, several milling parameters need to be adjusted, such as the ball-to-powder weight ratio, milling speed, and milling time. Milling time is a key parameter, and it directly affects the morphology and distribution of the milled powders. The relationships between the specific surface area of the powder and milling can be expressed as follows [13]:

(1) $$\mathrm{In}\frac{S_{m}-S_{0}}{S_{m}-S}=kt$$

Here, *k* is the constant of dispersion velocity, *t* is the milling time, and *S_m_* is the specific surface area of the material after ultimate milling, *S*_0_ is the specific surface area of the material before milling, and *S* is the specific surface area of the material after milling. Equation (1) revealed that a long milling time can refine the powders. However, it does not indicate that the powder can be incessantly crushed at an infinite milling time. With the extension of the milling time, the powder properties may deteriorate, including poor fluidity and agglomeration, due to the cold weld and work hardening. Meanwhile, large particles in powders can worsen the fluidity, compaction, and sinterability during sintering, ultimately affecting the properties of cermets. Besides, in the case of a milling time longer than the required time, the contamination in milled powders will increase. Hence, the powder should be appropriately milled to achieve a favorable composition, ensuring that the powder is in equilibrium between fracture and cold welding [14,15,16].

Thus, it is crucial to reveal the effect of milling times on the properties of milled powders and Mo_2_NiB_2_-Ni cermets. In this study, the microstructure and distribution of milled powders at different milling times were examined, as well as the mechanical properties of Mo_2_NiB_2_-Ni cermets prepared using milled powders at different milling times.

## 2. Materials and Methods

**Figure 1:**
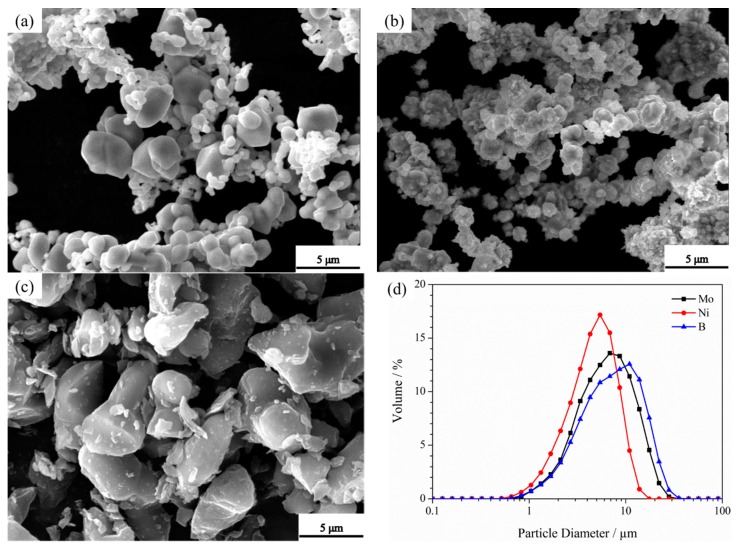
Micrographs of raw powders: (**a**) Mo, (**b**) Ni, and (**c**) B, and (**d**) their particle size.

Commercially available Mo, Ni, and B powders were used as raw materials. Table 1 and Figure 1 summarize the characteristics and microstructures of these powders, respectively. B exhibits an irregular morphology; meanwhile, Ni and Mo exhibit a near-spherical morphology. Table 2 summarizes the elemental compositions of the raw materials.

The raw powders were subjected to ball milling by using a planetary ball mill (Focucy, F-P400). To prevent the powders from oxidation during milling, a stainless-steel milling pot subjected to vacuum was used. A polytetrafluoroethylene (PTFE) liner was added in the pot, which can avoid damage from stainless steel during milling, caused by the ultrahard B powder. In addition, wet grinding with ethanol and a relatively low speed of 250 rotations per minute were employed to reduce the damage to the PTFE liner. Si_3_N_4_ grinding balls (Φ5 mm) with high hardness were used to insure that the ultrahard B powder can be crushed during ball milling at a ratio of 3:1 for the Si_3_N_4_ balls to the raw powders.

**Figure 2:**
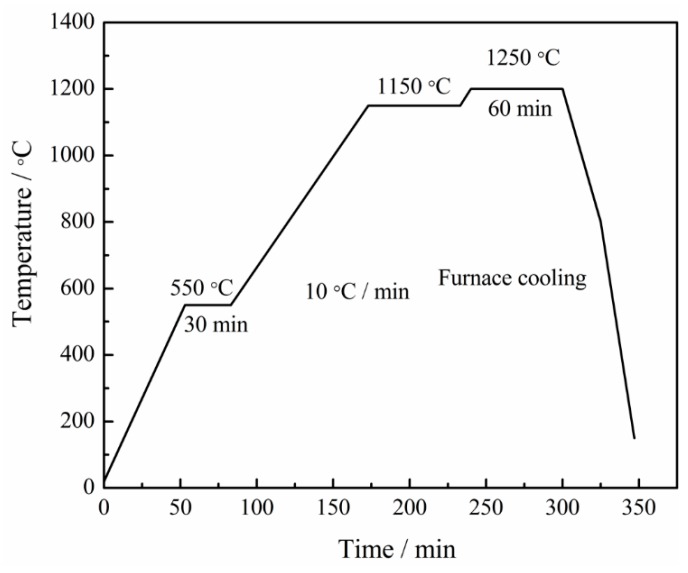
Sintering process of Mo_2_NiB_2_-Ni cermets.

The raw powders were milled at eight milling times, from 1 h to 15 h, with a constant increment of 2 h. The milled powder slurry was dried by using a rotary evaporator at 50 °C under vacuum. The milled powders were cold-isostatically shaped into cylindrical compacts (~Φ44 mm × 8 mm) under a pressure of 200 MPa at 120 s. The compacts were sintered in a vacuum furnace with graphite heaters (Chenhua, ZT-40, Shanghai, China), followed by placing them in a graphite crucible during the process. The furnace chamber was evacuated and remained to 10^−2^ Pa throughout the sintering process. During the heating process, the chamber was heated to 550 °C and preserved for 30 min to remove the PTFE in the compacts. After the heat preservation, the chamber was sequentially heated to 1200 °C at the heating rate of 10 °C/min and sintered for 60 min. The specimens were naturally cooled to room temperature inside the furnace. The master sintering curve was designed (Figure 2).

Scanning electron microscopy (SEM, Hitachi, SU3500, Tokyo, Japan), equipped with an energy-dispersive X-ray spectrometry (EDS), was employed to observe the microstructure of the milled powders and Mo_2_NiB_2_-Ni cermets. Electron Probe Microanalyzer (EPMA, JEOL, JXA-8230, Tokyo, Japan) was used to confirm the compositions of elements of the milled powders and Mo_2_NiB_2_-Ni cermets at different milling times. X-ray diffraction (XRD, Bruker, D8 Advance, Billerica, MA, USA) was employed to identify the phase compositions of the milled powders and Mo_2_NiB_2_-Ni cermets, and the results were obtained by using the XRD analysis software (MDI, Jade 6.0).

The relative densities and densities of Mo_2_NiB_2_-Ni cermets were estimated by a balance with a sensitivity of 0.01 mg at room temperature by the Archimedes’ method. In addition, the hardness, as Rockwell hardness (HRA), at room temperature was estimated. The Rockwell hardness tester (MX1000, Jinan, China) was utilized to estimate the hardness under a load of 50 kg and a dwell time of 10 s.

The three-point bending strength was estimated using a universal material testing machine (INSTRON, 1195, Boston, MA, USA). According to the test methods, the cermet specimens were cut into dimensions of 3 mm × 4 mm × 22 mm using 20-mm spans with a cross-head speed of 0.5 mm/min.

The results obtained from the above-mentioned properties corresponded to the statistical average of five specimens for each test to insure precision.

## 3. Results and Discussion

### 3.1. XRD Analysis of Powders at Different Milling Times

**Figure 3:**
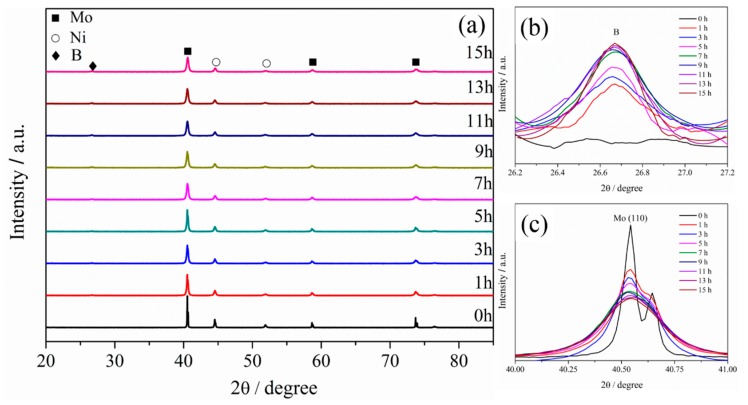
(**a**) XRD patterns of milled powders with milling from 0 h to 15 h, (**b**) evolution of peak of B with milling times, (**c**) evolution of (110) peak of Mo with milling times.

To analyze the change in the crystal structures of the milled powders during milling, XRD patterns of Mo, B, and Ni powders at different milling times were recorded with the increase in the milling time from 0 to 15 h. Figure 3a shows the XRD patterns. From the XRD patterns, diffraction peaks corresponding to Mo and Ni powders are clearly observed. Besides, a broad scattering peak with a relatively low intensity is observed at 26° (Figure 3b), and the intensity slightly increases with the increase in the milling time from 0 to 15 h, possibly related to the change in the brittle B powders to a non-crystallized powder by long-time milling. Moreover, new phases were not observed, and the diffraction peaks shifted with the increase in the milling time from 0 to 15 h, indicating that a solid solution does not form in the milled powders during milling. In addition, the result also revealed that mechanical alloying occurs by mechanical ball milling in this study. Furthermore, the XRD patterns revealed that the diffraction peaks of Mo and Ni become shorter and wider with the increase in the milling time from 0 to 7 h, which gradually change by the increase in the milling time from 7 h to 11 h. With the increase in the milling time to greater than 11 h, the diffraction peaks did not clearly change. In particular, with the increase in the milling time from 0 to 11 h, the height of the (111) peak of Mo (Figure 3c) sharply decreases, and the width rapidly increases and then slightly changes. In addition, from the XRD patterns, the full-width at half-maximum (FWHM) of the Mo and Ni diffraction peaks exhibits remarkable broadening, and the diffraction peak intensities of Mo and Ni are visibly weakened, mainly related to the distortion of the grain refining and lattice distortion caused by the ball-milling action. This phenomenon is in good agreement with the results reported by Sharafi et al. [17]. In this study, the decrease in the average crystallite size is typically reflected by the broadening of FWHM. Furthermore, according to the Williamson and Hall plot [18], the relationship between the crystallite size and FWHM can be concluded as follows:

(2) $$\frac{\beta^{2}}{\tan^{2}\theta }=\frac{K\lambda }{L}\cdot \frac{\beta }{\tan\theta \sin\theta }+16e^{2}$$

where *L* is the crystallite size, and *λ* is the FWHM, *K* is determined by the crystallite configuration. *β* is the spread that is related to the lattice strain and crystallite size alone. *e* is the wavelength of the X-ray radiation, and *θ* is the Bragg diffraction angle.

**Figure 4:**
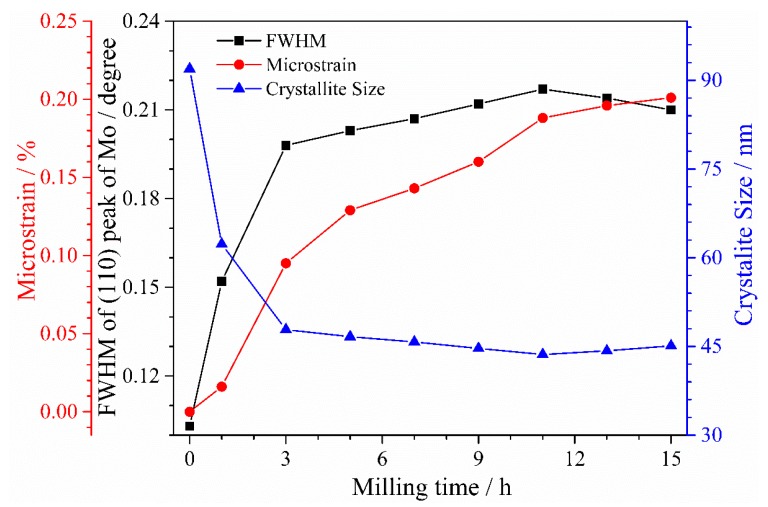
The full-width at half-maximum (FWHM), microstrain, and crystallite size of Mo with milling from 0 h to 15 h.

To further reveal the change in the microscopic crystal of the powder with the increase in the milling process from 1 h to 15 h, the FWHM of the (111) crystal plane and the average crystallite size of Mo (calculated by Equation (2)) are shown in Figure 4. The variation trend of FWHM revealed that with the increase in the milling time from 3 to 11 h, the FWHM of the Mo (111) peak rapidly increases from 1 to 3 h and then gradually increases (Figure 4). With the increase in the milling time to greater than 11 h, the FWHM of the Mo (111) peak slightly decreases. It also indicates that the milling time has a significant effect on the microstrain of Mo (110). The microstrain increases from 0.016% to 0.208% with the milling time increasing from 1 to 15 h, mainly due to the releasing of the microstrain of the refining powders and the deformation accumulation caused by impact energy during the milling process. From the variation trend of microstrain, it can be seen that although microstrain increases as the milling progresses, its effect on crystallite size is negligible. Meanwhile, the average crystallite size of the Mo powder changes with the opposite trend. With the increase in the milling time from 1 to 3 h, the average crystallite size of the Mo powder sharply decreases from 94.6 to 48.2 nm. With the increase in the milling time to greater than 3 h, the average crystallite size of the Mo powder continuously decreases, achieving the minimum value of 44.6 nm at a milling time of 11 h. However, with the increase in the milling time from 11 to 15 h, the average crystallite size of the Mo powder exhibits a slight upward trend. Moreover, the sharp decrease in the average crystallite size of the Mo powder with the increase in the milling time from 1 h to 3 h is related to the fact that the powders are severely crushed and broken by the high-speed grinding balls with a high collision energy. Furthermore, the average crystallite size of the Mo powder slightly decreases with the increase in the milling time from 3 h to 11 h due to the agglomeration of the powders caused by cold welding. In addition, the increase in the surface energy and plastic deformation of powders, which are caused by working hardening, may also make it difficult to further refine the powders. On the other hand, the more obvious effect of cold welding turning is the possible reason for the slight increase in the average crystallite size of the Mo powder with the increase in the milling time from 11 h to 15 h. Hence, the Mo powder exhibits the finest size at a milling time of 11 h, attributed to achieving the stable state between the powders of crush and cold welding.

### 3.2. EDS Analysis of Powders at Different Milling Times

**Figure 5:**
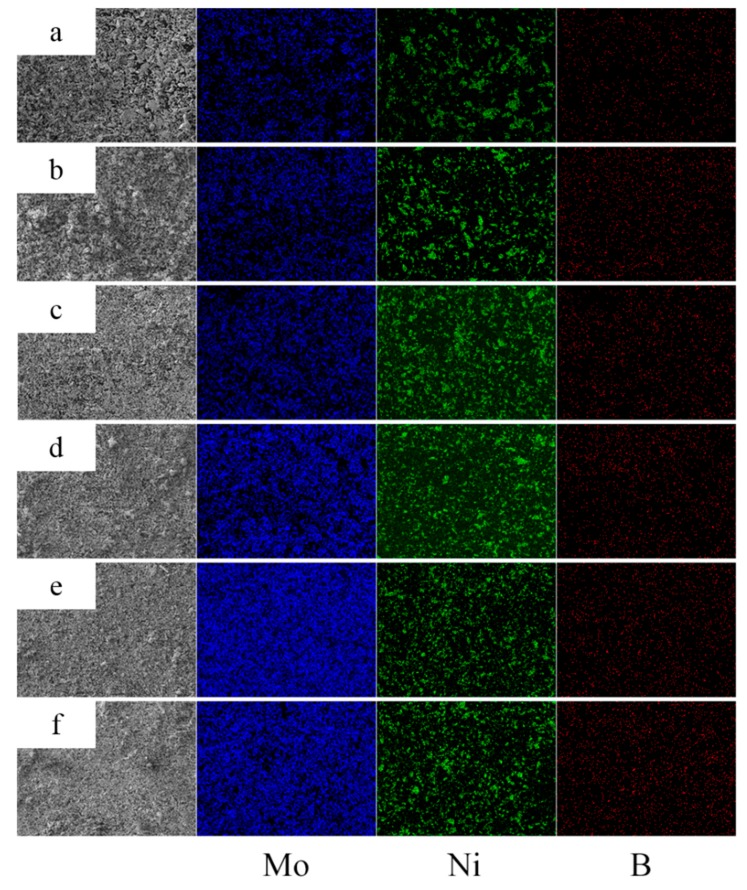
Energy-dispersive X-ray spectrometry (EDS) map analysis of the milled powders of milling times: (**a**) 0 h, (**b**) 1 h, (**c**) 3 h, (**d**) 7 h, (**e**) 11 h, and (**f**) 15 h.

To reveal the effect of the milling time on the distribution of the Mo, Ni, and B powders in milled powders, EDS mapping analysis was employed. Figure 5 shows the surface distributions of the Mo, Ni, and B elements in milled powders at milling times of 0 h, 1 h, 3 h, 7 h, 11 h, and 15 h. Blue, green, and red areas represent Mo, Ni, and B elements in Figure 5, respectively. With the increase in the milling time from 0 h to 3 h, clearly, the regions of the three colors were remarkably homogenized, indicating that the distributions of Mo, Ni, and B powders clearly improved, in addition to the ball milling. This phenomenon is probably related to the fact that raw powders are crushed into fragments under the impact of high-speed balls and are evenly mixed in the vial as a result of the movement of the balls. Moreover, the EDS results observed at 7 h and 11 h revealed that the blue and green regions are continuously homogenized with smaller particles than the previous time, indicating that Mo and Ni powders are not only more evenly distributed, but also gradually become finer. Meanwhile, the distribution of B powders does not exhibit any clear improvement with the increase in the milling time to greater than 3 h because it is difficult to crush the B powders and further make them thin due to their extremely high hardness, causing slight homogenization. Furthermore, as the milling time reaches 15 h, the improved homogenization of the Mo and Ni powders becomes less obvious than before as the regions of elements no longer tend to be homogenized, mainly because the homogenization of the milled powder achieves the limitation during milling.

Compositions of milled powders at different time is exhibited in Table 3 by using EMPA. Except Mo, Ni, and B elements, C, F, and Si elements in impurities also can be detected in the milled powders (not at 0 h). The source of C and F is the PTFE liner in the milling pot, which is peeled off during milling, related to the continuous impact of the ultrahard B powders and Si_3_N_4_ grinding balls. Meanwhile, Si elements originate from the damage of the Si_3_N_4_ grinding balls, which is related to the mutual impact between the grinding balls and the wear of the ultrahard B powders to the grinding ball. Moreover, with the increase in the milling time from 1 h to 15 h, the impurity content increases to some degree. In particular, with the increase in the milling time to greater than 11 h, the impurity content rapidly increases. Notably, to reduce the impurities, a relatively slow milling speed and a low ball-to-powder weight ratio are utilized for ball milling although this may lead to a lower limitation of size and the distribution of the milled powder.

### 3.3. Microstructure of Powders at Different Milling Times

**Figure 6:**
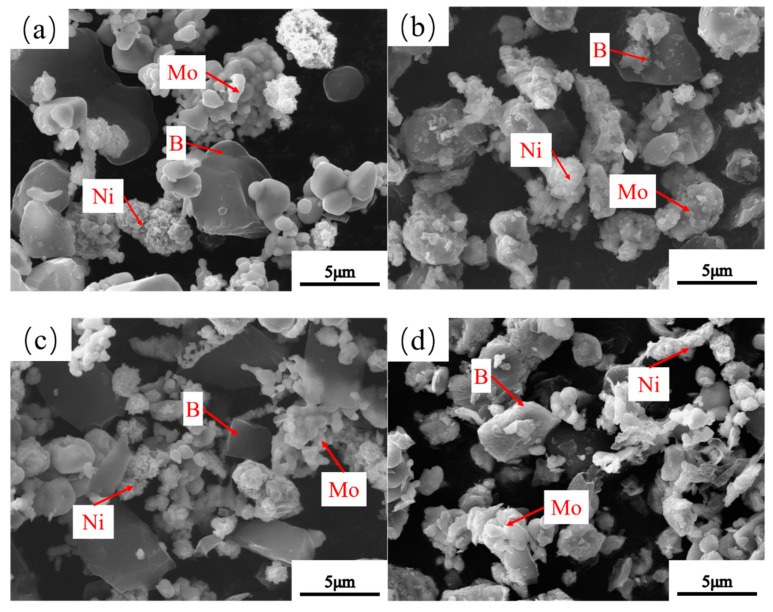

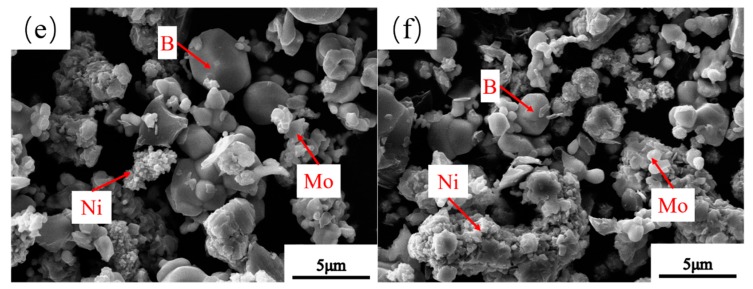
SEM pictures of milled powders of milling times: (**a**) 0 h, (**b**) 1 h, (**c**) 3 h, (**d**) 7 h, (**e**) 11 h, and (**f**) 15 h.

To investigate the effect of the milling time on the microstructure of the milled powders, the morphological images of the milled powders at milling times of 0 h, 1 h, 3 h, 7 h, 11 h, and 15 h were recorded (Figure 6). B powders exhibited irregular morphologies before milling, while Ni and Mo exhibit a globular morphology (Figure 6a). Moreover, the particles sizes of Mo, Ni, and B powders clearly decreased at the early stage of milling, with 1 h and 3 h (Figure 6b,c). Besides, the morphologies of B powders changed to a near-square morphology as the milling time reached 3 h, possibly related to the high brittleness of B powders. This brittleness in turn caused the B powders to be crushed to near-square fragments by the impact of hard Si_3_N_4_ grinding balls. Furthermore, with the increase in the milling time up to 7 h and 11 h (Figure 6d,e), Ni and Mo powders were continuously refined, mainly related to the occurrence of plastic deformation of the soft Mo and Ni powders caused by the sustained impact of high-speed grinding balls. Eventually, the big flakes in the powders were crushed into fragments, indicating that the milled powders become more brittle with the increase in the milling times from 3 h to 11 h. Besides, this phenomenon is consistent with the result reported by Ozkaya and Canakci [19]. At the initial stage of ball milling, the soft powders became brittle due to the accumulation of plastic deformation, and this transformation was mainly determined by the continuous impact between the grinding balls and powders. Meanwhile, the morphologies of B powders gradually tended to be near-spherical (Figure 6e), which is related to the continuous impact from the hard Si_3_N_4_ grinding balls. However, by comparing Figure 6e,f, the powders slightly changed as the milling time reached 15 h, as the powders getting the balance between crushing and cold welding due to the limitation of the relatively low ball-grinding speed and ball-to-batch ratio. In addition, Ni and Mo powder agglomerates were observed, which is consistent with the increase tendency of the average crystallite size in Figure 4.

**Figure 7:**
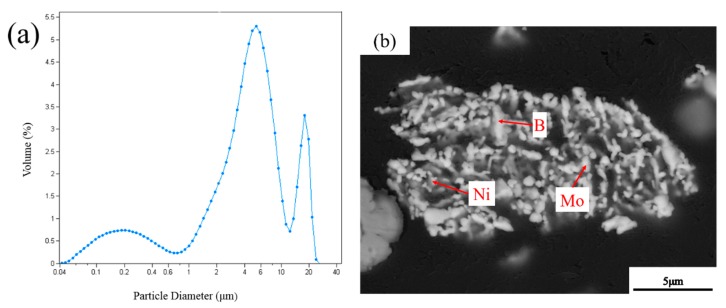
Particle size (**a**) and SEM picture of cross section (**b**) of milled powders at 11 h.

The laser particle sizer was utilized to further estimate the particle size of the milled powders at 11 h, and Figure 7a shows the result. The particle size mainly ranged between 3 μm and 10 μm. The particle size interval was considerably greater than the theoretical size of Mo powders calculated by Equation (2). To explain this anomaly, the morphology of the cross-section of milled powders at 11 h was observed (Figure 7b). This figure revealed that the particles of milled powders were aggregated from fine particles of Mo, Ni, and B powders. This agglomeration caused the experimental values for the powder size to be greater than the calculated ones. Ozkaya and Canakci [19] have reported that the properties of milled powders, including size, distribution, and morphology, crucially affect the microstructure and mechanical properties of the ceramics prepared by powder metallurgy. To epitomize, with the increase in the milling time from 1 to 7 h, the milled powders became finer in addition to the more uniform distribution. With the increase in the milling time to 11 h, the milled powders did not clearly exhibit further refinement. As the milling time reached 15 h, agglomeration was observed in the milled powders. Hence, a milling time of 11 h was selected as the optimum time in mechanical ball milling for the preparation of Mo_2_NiB_2_-Ni cermets.

### 3.4. Phase Composition of Mo_2_NiB_2_-Ni Cermets of Powders at Different Milling Times

**Figure 8:**
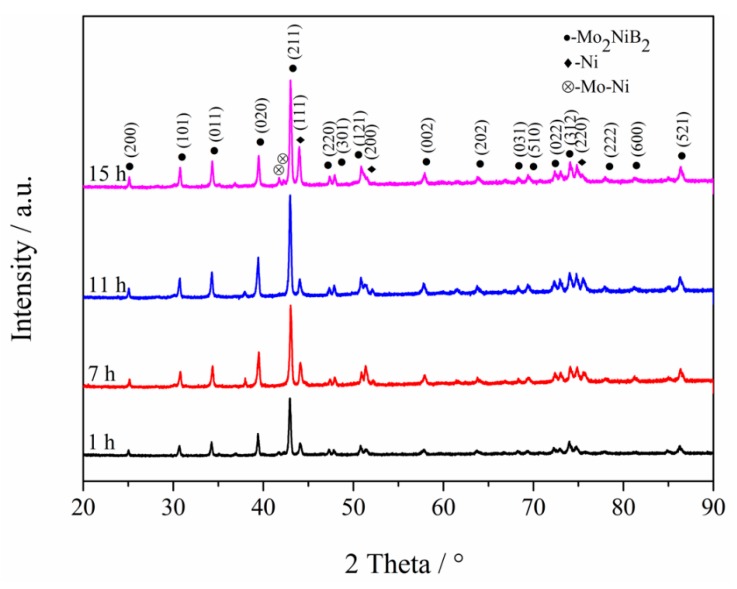
XRD patterns of Mo_2_NiB_2_-Ni cermets prepared by milled powders of four milling times.

To further investigate the impact of the milling time on the performance of Mo_2_NiB_2_-Ni cermets, bulks were prepared using milled powders at different milling times (1 h, 7 h, 11 h, and 15 h). XRD patterns were recorded to investigate the phase composition of the four cermets (Figure 8). From this figure, the Mo_2_NiB_2_ hard phase and Ni binder phase were detected in all of the four cermets. Moreover, with the increase in the milling time from 1 to 11 h, the peak height of the Mo_2_NiB_2_ phase, especially that of the (211) peak, clearly increased, indicative of a high peak intensity. Subsequently, the peak intensity of Mo slightly weakened as the milling time reached 15 h; this variation was possibly related to the incomplete generation of the Mo_2_NiB_2_ phase with the milled powders at milling times of 1 h and 7 h, due to the relatively small contact area between the powders as well as the uneven distribution. As the milling time reaches 11 h, the reactions for generating the Mo_2_NiB_2_ phase gradually became more consummate, attributed to the relatively large contact area between the fine milled powders in addition to the uniform distribution. Furthermore, with the continuous increase in the milling time to up to 15 h, the reactions were slightly restrained, due to the powder agglomeration. Hence, the reaction completion of the Mo_2_NiB_2_ phase reaction for the milled powders at a milling time of 15 h was worse than that at a milling time of 11 h. Besides, a small amount of the third phase was also detected, which was examined to be Mo-Ni (Figure 7). Yuan et al. [14] reported that the Mo_2_NiB_2_ hard phase is formed before the liquid phase by the reaction Mo + B = MoB and later by 2MoB + Ni = Mo_2_NiB_2_. However, the formation mechanism of the Mo_2_NiB_2_ phase at temperatures greater than 900 °C is 2Mo + Ni + 2B + Mo_2_NiB_2_ = 2Mo_2_NiB_2_, where the previously formed Mo_2_NiB_2_ particles act as seed crystals, allowing Mo, Ni, and B elements to directly produce new particles. Based on the above findings, the Mo-Ni phase is not generated with the formation of the Mo_2_NiB_2_ phase. Kubliy et al. [13] have reported that the Mo–Ni phase is produced by the dissolution of Mo in Ni at the Ni-rich region between 1160 °C and 1231 °C, which is consistent with the extensive homogeneity region of the nickel-based solid solution in a Mo-Ni binary system. This theory is consistent with the variation trend of the peak intensity of Mo-Ni, which gradually decreases in addition to the more uniform distribution of milled powders with the increase in the milling times from 1 to 11 h. The uniform distribution of milled powders can reduce the high Ni content region during sintering. On the other hand, the Ni-rich regions may also be formed during the sintering of the cermets with milled powders at 15 h, due to the agglomeration as a result of long-time milling.

**Figure 9:**
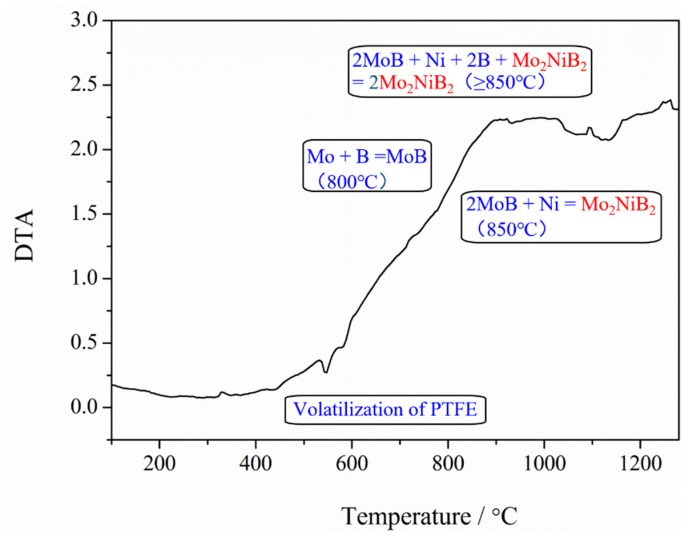
Differential thermal analysis (DTA) cover of milled powders from 100 to 1250 °C under Ar atmosphere.

To further confirm the phase formation during sintering, DTA analysis was employed using the milled powders heated to 1250 °C under Ar at 10 °C/min. The curve is relatively flat at temperatures ranging from 100 to 700 °C (Figure 9). A weak endothermal peak is observed at 520 °C, corresponding to the volatilization of a small amount of PTFE in milled powders. At temperatures greater than 800 °C, a wide exothermic peak is observed, which is consistent to the study reported by Bo Yuan. This peak revealed that raw powders start to react at 800 °C. Moreover, an endothermal peak is observed at 1100 °C; this temperature is in accordance with that of the liquid phase observed according the study reported by Takagi. K.

### 3.5. Microstructure of Mo_2_NiB_2_-Ni Cermets of Powders at Different Milling Times

**Figure 10:**
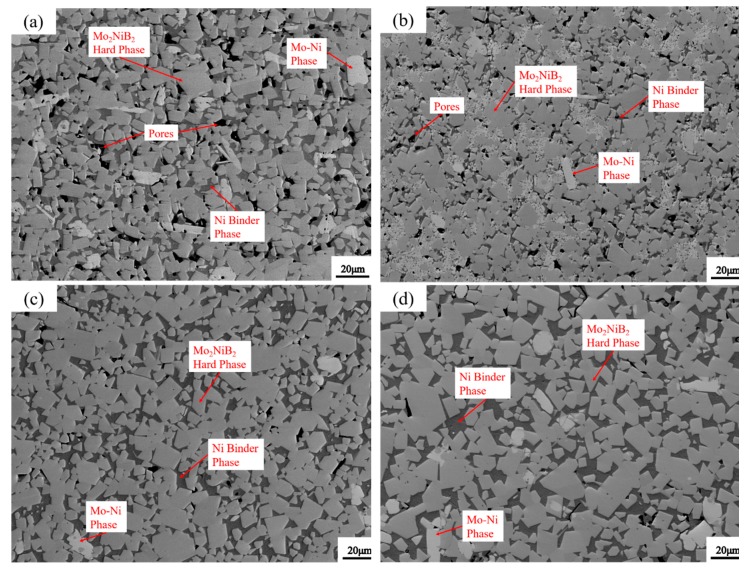
SEM pictures of Mo_2_NiB_2_-Ni cermets prepared by milled powders of four milling times: (**a**) 1 h, (**b**) 7h, (**c**) 11 h, and (**d**) 15 h.

Figure 10 shows the SEM photographs of the Mo_2_NiB_2_-Ni cermets prepared from milled powders at milling times of 1 h, 7 h, 11 h, and 15 h. Three phases are clearly observed in this figure: The grey and dark phases are the Mo_2_NiB_2_ hard phase and the Ni binder phase, respectively. Meanwhile, the few white third phases are also confirmed to be the Mo-Ni phase. In addition, voids and cracks are clearly observed in specimens at milling times of 1 h and 7 h (Figure 10a,b). The possible reason for the defects could be that, during liquid-phase sintering, the densification of cermets mainly depends on the liquid-phase flow and particle rearrangement. However, the interdiffusion in the liquid phase is limited due to the non-uniform distribution as well as the large particles in the milled powders at milling times of 1 h and 7 h. Besides, the complete liquid-phase sintering process depends on the wettability and solubility between the milled powders and the liquid phase, and these two points are related to the size of the milled powders. According to the Young equation [13], only $\gamma_{SL}<\gamma_{L}+\gamma_{S}$ ($\gamma_{SL}$ is the interfacial energy between the solid and liquid phases, $\gamma_{L}$ and $\gamma_{S}$ are the specific surface energies of the liquid and solid phases, respectively), the wettability of the liquid phase with solid particles is good. Thus, the wettability can be improved in the case of finer powders, attributed to the decrease of $\gamma_{SL}$. Moreover, the relationship between the solubility of particles and their size can be expressed as follows [13]:

(3) $$\Delta L=\frac{2\gamma_{SL}\delta^{3}}{kT}\cdot \frac{1}{r}\cdot L_{\infty }$$

Here ΔL is the saturated solubility, $\gamma_{SL}$ is the interfacial energy, *T* is the temperature, and *r* is the particle radius.

Equation (3) revealed that the saturation solubility is inversely proportional to the particle radius. Hence, the solubility increases with the fine powder. In other words, the relative density of Mo_2_NiB_2_-Ni increases along with the finer milled powders. Hence, voids in the specimens with powders milled at a milling time of 11 h (Figure 10c) clearly decrease as the interdiffusion enhanced during sintering with the powders becomes fine and uniform. Hence, the densification of the specimen at a milling time of 11 h remarkably increases, with barely any defects. Furthermore, by comparing Figure 10d with Figure 10c, the grain growth of Mo_2_NiB_2_ is clearly significant as the milling time reaches 15 h, but the specimen is not densified further. The reason for the merging and growing of Mo_2_NiB_2_ grains is that the high surface energy and the relatively larger area of contact between the powders caused by long-time balling limit the viscous flow of the liquid phase and shrinkage of voids.

**Figure 11:**
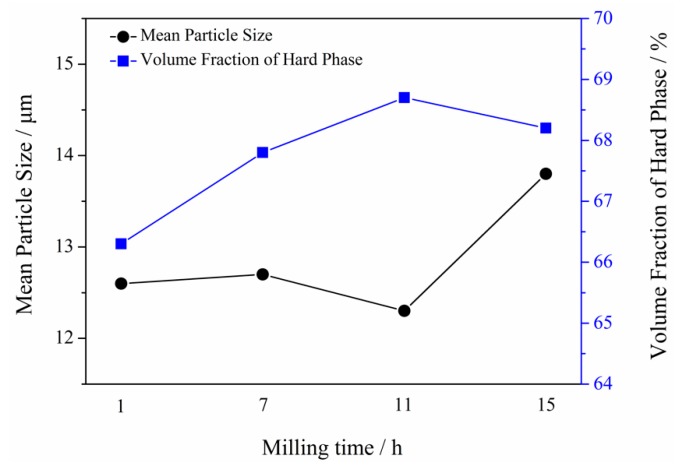
The morphology evolution of Mo_2_NiB_2_-Ni cermets prepared by milled powders of four milling times.

To further investigate the effect of the milling time on the microstructure of Mo_2_NiB_2_-Ni cermets, the volume ratio and grain size of the Mo_2_NiB_2_ hard phase in specimens at different milling times were estimated (Figure 11). The figure revealed that the volume fraction of the Mo_2_NiB_2_ hard phase significantly increases with milled powders with the increase in the milling time to 11 h, followed by a slight decrease with the increase in the milling time up to 15 h. In addition, the volume fraction of the hard phase reaches the maximum with milled powders at a milling time of 11 h, which is consistent with the change in the peak intensity of the Mo_2_NiB_2_ phase in the above-mentioned XRD analysis results of cermets. Meanwhile, the grain size of the Mo_2_NiB_2_ hard phase gently decreases with milled powders with the increase in the milling time from 1 h to 7 h and then clearly decreases with the increase in the milling time from 7 to 11 h. However, the grain size significantly increases as the milling time reaches 15 h. The possible reason for these variation trends is that the specific surface area of the powders increases with the refining, and the wettability and solubility of the milled powders in the liquid phase are simultaneously improved. Hence, reactions are sufficiently carried out during the solution–precipitation stage, leading to the increase in the volume fraction of the Mo_2_NiB_2_ hard phase. In addition, with the more adequate reactions of the Mo_2_NiB_2_ hard phase, more crystal nuclei of Mo_2_NiB_2_ are formed in the liquid phase. Thus, the size of Mo_2_NiB_2_ particles reaches the minimum with cermets prepared using powders at a milling time of 11 h.

**Figure 12:**
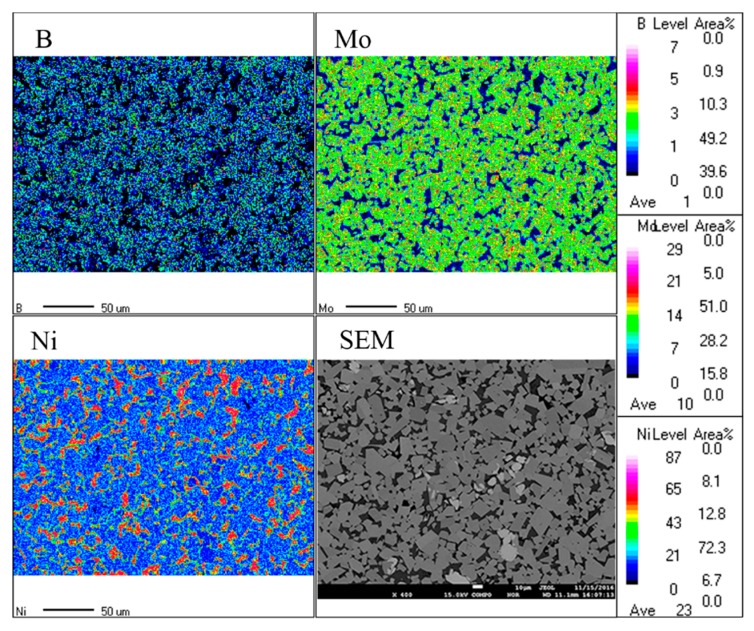
Electron probe microanalyzer (EPMA) map analysis of Mo_2_NiB_2_-Ni cermets prepared by milled powders of milling 11 h.

Figure 12 shows the EPMA map analysis of Mo_2_NiB_2_-Ni cermets prepared using milled powders at a milling time of 11 h, indicative of the elemental distribution in cermets. The distributions of the Mo_2_NiB_2_ hard phase and the Ni binder phase in the cermets are clearly uniform (Figure 12). Moreover, the chemical constituents and elemental composition of different phases in the specimen are confirmed by EPMA point analysis. Table 4 summarizes the results obtained: The atomic ratio of Mo, Ni, and B in the Mo_2_NiB_2_ hard phase is ~2:1:2, which is consistent with the theoretical composition of the Mo_2_NiB_2_ hard phase. Besides, few Mo elements are detected in the Ni binder phase due to the interdiffusion of elements through a liquid phase during solid-phase dissolution–reprecipitation. In addition, the elemental composition of the third phase is further confirmed by the above analysis as the Mo-Ni phase is formed by the dissolution of Mo in Ni.

### 3.6. Mechanical Properties of Mo_2_NiB_2_-Ni Cermets of Powders at Different Milling Times

**Figure 13:**
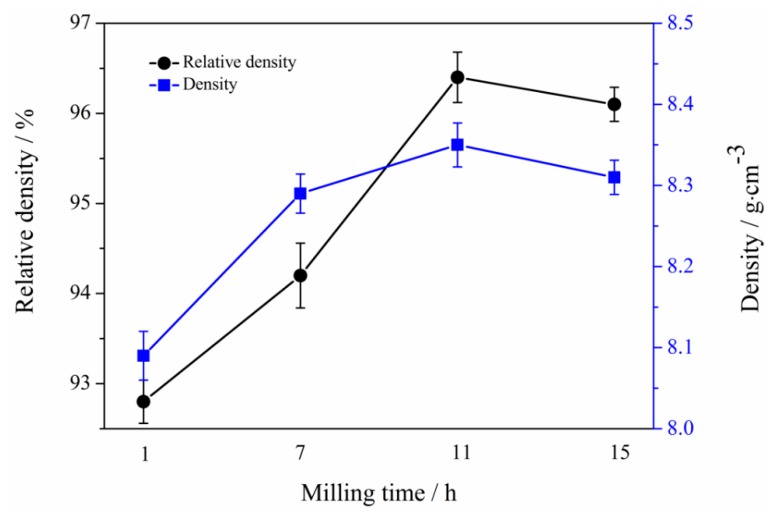
Density and relative density of Mo_2_NiB_2_-Ni cermets prepared by milled powders of four milling times.

Figure 13 shows the densities and relative densities of the specimens prepared using milled powders at milling times of 1 h, 7 h, 11 h, and 15 h. Clearly, the density and relative density of Mo_2_NiB_2_-Ni cermets prepared using milled powders at milling times of 1 h and 7 h are relatively low because large, nonuniform powders limit the interdiffusion in the liquid phase, leading to a slow liquid-phase flow and incomplete particle rearrangement. Moreover, the densities and relative densities of the cermets are clearly high for powders milled at a milling time of 11 h. On the other hand, the density and relative density slightly decrease as the milling time reaches 15 h, which is related to the limitation of the viscous flow of the liquid phase and shrinkage of voids by large Mo_2_NiB_2_ grains.

**Figure 14:**
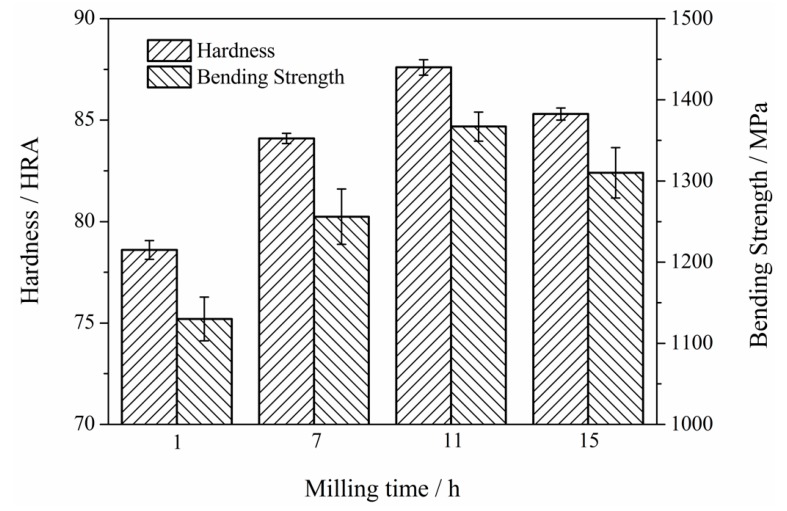
Mechanical properties of the Mo_2_NiB_2_-Ni cermets prepared by milled powders of four milling times.

The mechanical properties, such as the hardness and bending strength of Mo_2_NiB_2_-Ni cermets prepared using milled powders at different milling times, are measured. Figure 14 shows the results obtained. The figure indicated that the hardness clearly increases with the increase in the milling time from 1 h to 11 h. Subsequently, the hardness slightly decreases as the milling time reaches 15 h. In addition, the variation trend of the bending strength of Mo_2_NiB_2_-Ni cermets with the milling time is similar to the trend of hardness. Moreover, the trends of the mechanical properties revealed that cermets prepared using milled powders at a milling time of 11 h exhibit the best mechanical properties. In other words, the hardness and bending strength of the cermets at a milling time of 11 h reach the maximum, possibly related to the fact that the relative density of the specimen at a milling time of 11 h is clearly improved in comparison with that of the specimen at milling times of 3 h and 7 h (Figure 10). However, the two properties deteriorate as the milling time reaches 15 h, and this observation is consistent with the growth of the grains for the specimen milled at a milling time of 15 h (Figure 10c).

**Figure 15:**
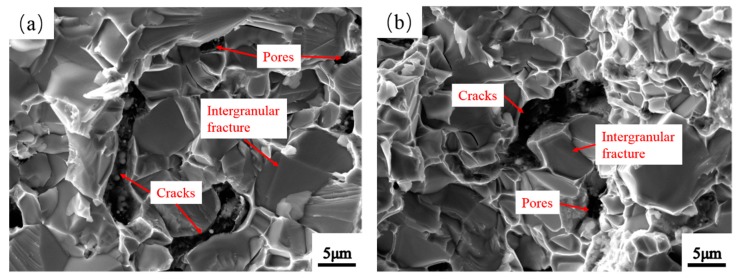

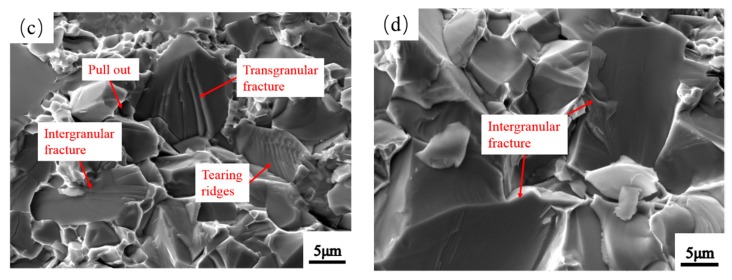
Fracture surface f Mo_2_NiB_2_-Ni cermets prepared by milled powders of four milling times: (**a**) 1 h, (**b**) 7 h, (**c**) 11 h, and (**d**) 15 h.

Figure 15 shows the fracture morphologies of Mo_2_NiB_2_-Ni cermets at four milling times. Cracks and pores are clearly observed on the fracture surface (Figure 15a,b). The main fracture mode is brittle fracture with an intergranular fracture. Moreover, intergranular and transgranular fractures are clearly observed on the fracture surface of cermets at a milling time of 11 h (Figure 15c). In addition, tearing ridges are observed (Figure 15c). In addition, a small amount of pull out and debonding is observed (Figure 15c), indicative of good bending strength. Furthermore, the intergranular fracture of abnormal big Mo_2_NiB_2_ particles is observed with no voids (Figure 15d).

## 4. Conclusions

(1) A new phase or a mechanical alloy is not detected during the milling of Mo, Ni, and B powders. The milled powders are crushed to small fragments, in addition to the more uniform distribution with the increase in the milling time from 1 h to 11 h; however, the milled powders slightly change with the increase in the milling time to 11 h.

(2) Mo_2_NiB_2_-Ni cermets are successfully prepared using milled powders at different milling times, while there is a small amount of the Mo-Ni phase precipitation in specimens milled at milling times of 1 h, 7 h, and 15 h due to the nonuniform distribution and agglomeration of milled powders. Meanwhile, the cermets prepared using milled powders at a milling time of 11 h exhibit optimal morphology with an optimum relative density and the smallest crystal size of Mo_2_NiB_2_.

(3) The mechanical properties, such as the hardness and bending strength, exhibit the maximum value for Mo_2_NiB_2_-Ni cermets prepared using milled powders at a milling time of 11 h, of 87.6 HRA and 1367.3 MPa, respectively.

## Figures and Tables

**Table 1:** Characteristics of Mo, Ni, and B powders.

Powder	Chemical Composition (wt%)	Particle Size (μm)	Manufacturer
Mo	Fe < 0.005, Si < 0.002, Mg < 0.002	3.4–8.6	Changsha Tianjiu Metal Material Corp., Ltd., Changsha, China
Ni	Fe < 0.006, Mg < 0.002	2.8–6.8	Changsha Tianjiu Metal Material Corp., Ltd., Changsha, China
B	Fe < 0.005, Mn < 0.003	5.4–10.1	Baoding Zhongpuruituo Technology Corp., Ltd., Baoding, China

**Table 2:** Compositions (wt%) design of the milled powders in this study.

Mo	Ni	B
60.5	33.5	6.0

**Table 3:** Compositions of the milled powders at different milling times.

Milled Powders	Compositions (wt%)					
	Mo	Ni	B	C	F	Si
0 h	61.26	33.02	5.72	-	-	-
1 h	60.50	32.66	5.48	1.36	-	-
3 h	57.43	30.24	5.20	4.28	1.17	1.68
7 h	56.75	28.64	5.15	6.07	1.52	1.87
11 h	55.86	27.21	5.07	7.62	2.18	2.06
15 h	53.97	25.83	4.69	10.04	3.16	2.31

**Table 4:** Composition analysis of the three different phases in Figure 12.

Point	Composition (at%)		
	Mo	Ni	B
1	40.31	21.36	38.33
2	3.98	96.02	-
3	64.19	35.81	-
